# Clinical impact and therapeutic potential of tertiary lymphoid structures in melanoma

**DOI:** 10.3389/fimmu.2026.1826453

**Published:** 2026-08-03

**Authors:** Daniele Carenza, Chiara Bungaro, Michele Guida, Benedetta Apollonio

**Affiliations:** Rare Tumors and Melanoma Unit, IRCCS Istituto Tumori “Giovanni Paolo II”, Bari, Italy

**Keywords:** germinal center, immunotherapy, melanoma, prognostic markers, tertiary lymphoid structures

## Abstract

Tertiary lymphoid structures (TLS) are ectopic lymphoid aggregates that arise in non-lymphoid tissues under conditions of chronic inflammation, including cancer. Structurally and functionally resembling secondary lymphoid organs, TLS consist of organized T-cell zones, B-cell follicles, dendritic cells, high endothelial venules, and specialized stromal cells, enabling local antigen presentation and adaptive immune activation. In melanoma, the prototypical immunogenic tumor, the presence of TLS within the tumor microenvironment has been associated with enhanced immune surveillance and favorable clinical outcomes. However, emerging evidence indicates that TLS can also harbor immunosuppressive niches, potentially depending on their developmental stage and cellular composition. In this review, we summarize the molecular and cellular mechanisms underlying TLS biogenesis and outline the experimental methodologies currently used for their identification and characterization, with a focus on melanoma. We examine the clinical relevance of TLS in both primary and metastatic melanomas and discuss strategies aimed at inducing functional TLS within tumors. By promoting TLS formation to amplify intratumoral immune responses, these approaches hold promise for overcoming resistance to immunotherapy and improving outcomes in advanced melanoma and other malignancies.

## Introduction

Tertiary lymphoid structures (TLS) are organized aggregates of immune cells that arise in non-lymphoid tissues during chronic inflammatory conditions. They have been described in a variety of pathological conditions, including primary or metastatic tumor sites, autoimmune-affected tissues, sites of graft rejection, and infected tissues (1–3). Depending on the context, TLS can either promote or impede disease evolution. In autoimmune diseases, chronic inflammation, and ageing, the presence of TLS correlates with a more severe disease course (4). In cancer, TLS display functional heterogeneity, exerting context-dependent effects on tumor immunity. They may either foster the establishment and maintenance of an immunosuppressive tumor microenvironment (TME), that constrains effective antitumor immune responses, or promote productive antitumor immunity and enhance responsiveness to immunotherapy (5).

Melanoma represents a particularly compelling context in which to investigate TLS biology. It is highly immunogenic, characterized by high tumor mutational burden (TMB), abundant neoantigen generation, and a well-documented capacity to elicit spontaneous adaptive immune responses (6, 7). These properties favor an immunological milieu supportive TLS neogenesis, and indeed, TLS have been identified across primary and metastatic melanoma lesions. However, melanoma is also a disease of profound immunological heterogeneity, as its major histological subtypes differ in their mutational landscapes, stromal architecture, and immune infiltration patterns. These differences are reflected in subtype-specific variation in TLS prevalence, maturation state, and prognostic relevance. Despite growing interest, several critical gaps remain to be addressed. Recent studies have examined TLS across solid tumors and established generalizable principles of TLS biology (2, 5, 8, 9). Melanoma-specific considerations, including TLS identification strategies, subtype-dependent TLS heterogeneity, spatial and functional divergence between primary and metastatic TLS, and organ-specific variation in TLS formation across metastatic sites, have not been systematically addressed. Furthermore, the translational bottleneck separating mechanistic TLS biology from actionable therapeutic strategies remains largely uncharted. Key unresolved TLS-related questions include the need for standardized and clinically applicable quantification methods, the validation of TLS-inducing therapeutic strategies, and a clearer understanding of how TLS biology intersects with mechanisms of resistance to immune checkpoint inhibitors (ICIs).

Here we summarize the current literature on TLS biology in melanoma, and cover identification methodologies, subtype-specific heterogeneity, spatial distribution across primary and metastatic lesions. We examine how maturation state and intratumoral localization shape prognostic significance, discuss TLS as predictive biomarkers of ICI response, and explore preclinical and clinical strategies aimed at inducing or enhancing TLS formation to potentiate antitumor immunity.

## TLS structure and neogenesis

Although a universally accepted system for identifying and staging has not yet been established, TLS are typically classified into two major maturation states based on their morphological and functional features: immature TLS (iTLS), and mature TLS (mTLS) (5, 10).

iTLS are characterized by aggregates of B and T lymphocytes that lack well-defined spatial segregation and germinal center (GCs)–specific stromal cells, such as follicular dendritic cells (FDCs). Although it remains unclear whether iTLS represent an early stage of TLS maturation, their presence in certain cancer types has been associated with T-cell exhaustion, and their ‘immaturity’ correlates with immunosuppression rather than enhanced antitumor immune responses (11).

**Figure 1:**
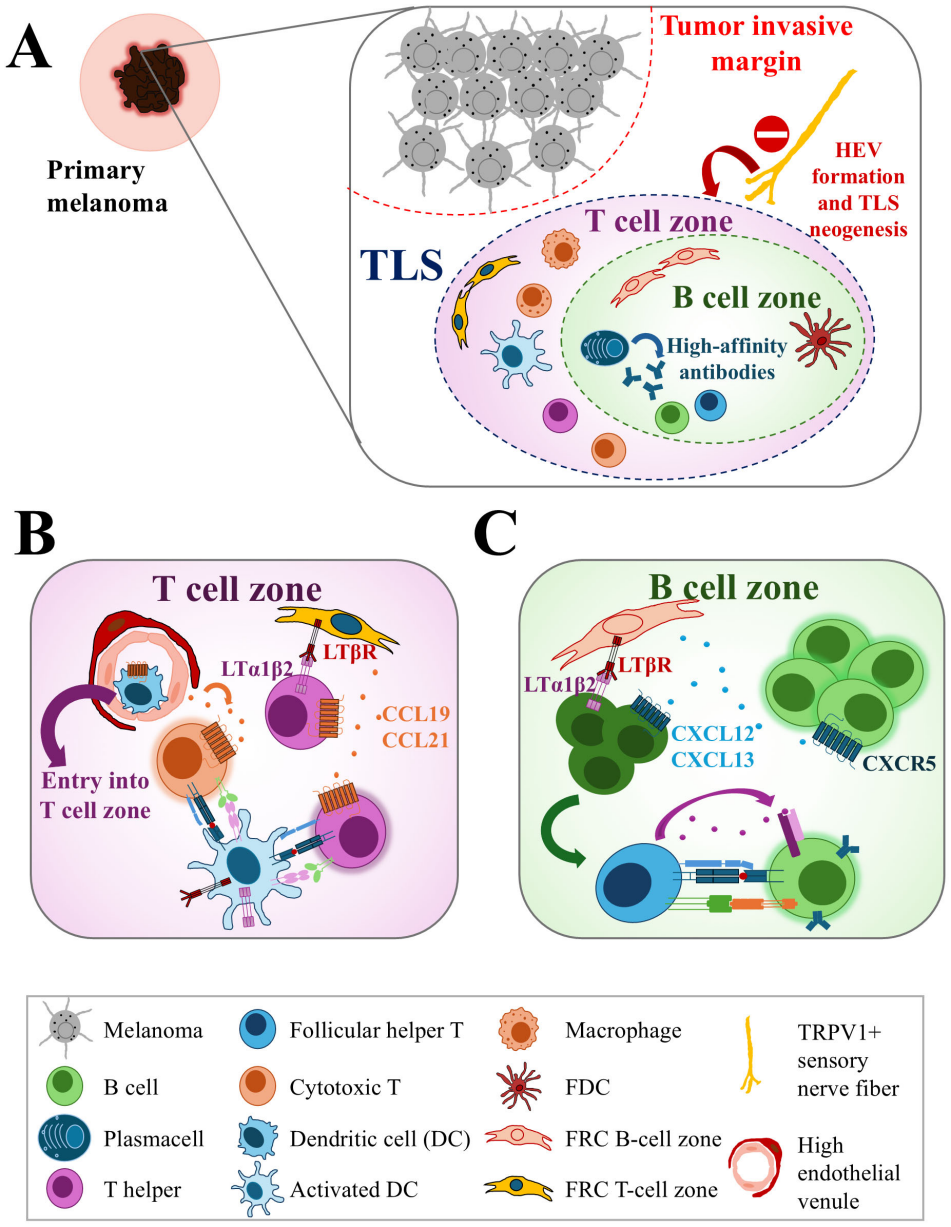
Cellular interactions within tertiary lymphoid structures (TLS) in the peritumoral region of cutaneous melanoma. Cartoon illustrating the intercellular interactions occurring in a mTLS. **(A)** Overview of the TLS cellular composition, highlighting the compartmentalization of B- and T-cell zones. A peripheral TRPV1^+^ sensory nerve fiber is shown inhibiting high endothelial venule (HEV) formation, TLS-specific chemokine secretion, and TLS neogenesis. **(B)** T-cell zone. Lymphoid tissue inducer (LTi)– lymphoid tissue organizer (LTo) interactions sustain TLS architecture and facilitate progressive activation of CD4^+^ and CD8^+^ T cells. Mature dendritic cells (DCs) present processed melanoma-specific or melanoma-associated antigens to naïve T cells, thereby priming melanoma-reactive T-cell clones. Co-stimulatory signaling via CD80/CD86–CD28 enhances T-cell activation. Upon activation, T cells downregulate CCR7, migrate toward the tumor bed, and exert antitumor effector functions. CCR7^+^ mature DCs and T cells are recruited into the T-cell zone by stromal cells expressing CCL19 and CCL21. Similar to lymph nodes, leukocytes gain access to TLS through HEVs. **(C)** B-cell zone. LTi–LTo interactions contribute to TLS structural maintenance and support B-cell activation. B cells receive further stimulation from colocalizing T follicular helper (Tfh) cells. Stable contact is mediated by CD40–CD40L interaction, while IL-21 secreted by Tfh cells binds to IL-21R on B cells, promoting immunoglobulin class switching and somatic hypermutation, which are essential for antitumor immunoglobulin production. CXCR5^+^ B cells are recruited to the B-cell zone by CXCL12 and CXCL13 produced by stromal cells.

mTLS comprise structures containing either primary follicles lacking GCs or secondary follicles with well-developed GCs. Distinct stages of TLS maturation can coexist within the same tissue, suggesting that local heterogeneity in the TME may influence TLS maturation or that TLS could arise at different time points (12). mTLS are characterized by well-defined compartmentalization, with a centrally located B-cell zone surrounded by a peripheral T-cell zone. This maturation stage features B-cell zone gathered CD21^+^ FDCs and CD4^+^ PD-1^+^ ICOS^+^ CXCR5^+^ T follicular helper (Tfh) cells (**Figure 1**).

TLS neogenesis shares core developmental features with secondary lymphoid organ formation but remains distinct in structural organization, anatomical integration, and functional output. Although the mechanisms governing TLS initiation and maturation are incompletely defined, current evidence has identified key cellular and molecular pathways.

Within the TME, several immune populations can acquire lymphoid tissue–inducer (LTi) functions, including type 3 innate lymphoid cells, effector CD8^+^ T cells, T helper -17 cells, natural killer cells, B cells, dendritic cells (DCs), and M1 macrophages (5). Lymphoid tissue–organizer (LTo) activity is provided by stromal and endothelial cells, with fibroblasts acting as central drivers of TLS neogenesis (13, 14). In murine models, immunofibroblasts are sequentially activated by IL-13 and IL-22 and subsequently sustained by lymphocyte-derived signals, giving rise to a stromal network resembling fibroblastic reticular cells in secondary lymphoid organs (15). Distinct fibroblast subsets support B- and T-cell compartmentalization through expression of lymphoid chemokines and survival factors.

TLS induction critically depends on LTα1β2–LTβR signaling between LTi and LTo cells, which activates NF-κB–dependent expression of CCL19, CCL21, and CXCL13, promoting immune cell recruitment and reinforcing TLS development (**Figure 1**) (16). In melanoma models, PDPN^+^ FAP^-^ cancer-associated fibroblasts act as surrogate LTo cells, highlighting fibroblast heterogeneity in TLS formation (17). As in secondary lymphoid organs, lymphocyte entry into TLS occurs via high endothelial venules (HEV), whose maturation and maintenance are regulated by TNFR and LTβR signaling (18); disruption of this pathway impairs HEV formation and TLS architecture (19).

A new interaction regulating TLS development has also been described, and it involves the peripheral nervous system. Sensory nervous fibers innervating skin have been shown to sustain immunosuppressive TME, hinder TLS neogenesis, and indirectly promote solid tumor progression (**Figure 1**). Their ablation promotes iTLS neogenesis, HEVs maturation, and improved leukocyte recruitment, adding another layer of complexity to the regulation of TLS neogenesis (20).

## TLS in solid tumors

TLS have been identified in several solid tumors beyond cutaneous melanoma, where they frequently correlate with favorable clinical outcomes. In non-small cell lung cancer (NSCLC), high intratumoral TLS count is associated with improved overall, disease-specific, and recurrence-free survival (21, 22). TLS^+^ NSCLC exhibit distinct immune checkpoint expression profiles and reduced immune cell dysfunction compared with TLS^-^ tumors, suggesting that TLS may serve as predictive biomarker of immunotherapy response (22). A similar prognostic role has been reported in colorectal carcinoma (CRC), where TLS-related signatures predict immune checkpoint expression and may help to identify patients that could benefit from immunotherapy (23, 24). On the other side, the prognostic significance of TLS in hepatocellular carcinoma (HCC) remains controversial. Early hepatic lesions predominantly contain iTLS, which are associated with enhanced antigen presentation and active antitumor immunity on a background of elevated expression of immune checkpoints and immunosuppressive cytokines (25). In contrast, mTLS positively correlate with overall and recurrence-free survival in both lymphocyte-rich and conventional HCC (26). In high-grade serous ovarian cancer, TLS are more frequently observed in the Fallopian tubes than in the ovaries, where they display transcriptional programs linked to B-cell activation and humoral antitumor responses (27). In adult-diffuse gliomas, TLS maturation is characterized by plasma cell abundance, B-cell activation, and B/T-cell interactions (28). Gliomas enriched in immunoglobulin-producing plasma cells and activated B-cells exhibit significantly longer overall survival (OS) (28).

Evidence from multiple solid tumors highlights the importance of TLS composition, maturation state, and anatomical location in shaping antitumor immunity and clinical outcomes. Nevertheless, a comprehensive discussion of TLS across cancers other than cutaneous melanoma is beyond the scope of this work.

## Tools for TLS identification

TLS can be identified using different experimental strategies, including hematoxylin-eosin staining (H&E), immunohistochemistry (IHC), immunofluorescence (IF), spatial transcriptomics, spatial proteomics, and multi-omics approaches.

The tumoral region where TLS can be identified must be carefully selected, especially when the procedure can be affected by operator subjectivity. For this reason, necrotic and ulcerated areas must be excluded during TLS identification (29).

**Figure 2:**
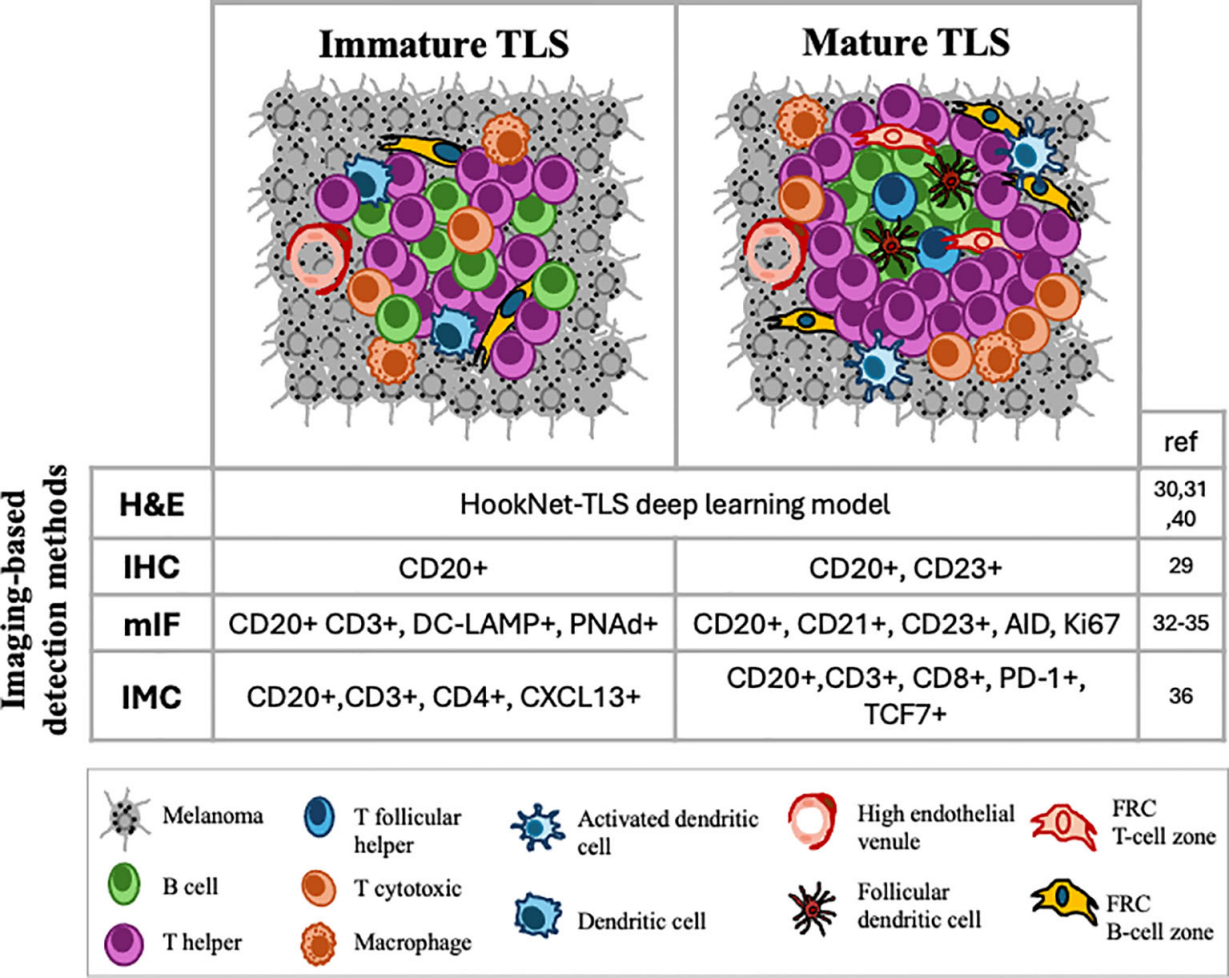
Immature versus mature TLS in primary cutaneous melanoma. H&E, hematoxylin-eosin, IHC, immunohistochemistry, mIF, multiplex immunofluorescence, IMC, imaging mass cytometry. The HookNet-TLS algorithm integrates multiple image resolutions from H&E-stained whole-slide images to identify both immature (iTLS-GC-negative) and mature (mTLS-GC-positive) TLS. TLS maturation can also be evaluated by IHC: CD20 single staining identifies B-cell clusters within iTLS, whereas CD20/CD23 double staining detects B-cell follicles and GC-associated FDCs within mTLS. Higher-resolution spatial characterization is achieved by mIF, revealing disorganized CD20^+^ B cells and CD3^+^ T cells, potentially reaching the TME via HEVs (PNAd^+^), interacting with antigen-presenting DCs (DC-LAMP+) in iTLS. In mTLS, proliferating B cells (CD20^+^ Ki67^+^) and T cells (CD3^+^ Ki67^+^) segregate in distinct compartments with GC-associated stromal cells (CD21^+^ CD23^+^) sustaining class-switched B cell (CD20^+^ AID^+^) functions. IMC enables simultaneous analysis of up to 50 protein markers, allowing comprehensive characterization of immune cell subsets and functional states, cytokine distribution within the TME and the immunomodulatory roles of immune populations across immature and mature TLS.

H&E is the most cost-effective histopathological technique, it is easy, with rapid turnaround time, and has broad clinical applicability. Despite these advantages, H&E-based analyses exhibit lower specificity compared with more advanced image-based approaches (16). Consequently, reliable TLS identification on H&E slides depends on meticulous morphological evaluation. Key features include lymphocyte nuclear morphology, cellular density, and relative nuclear volume compared with adjacent tissue components. In this context, the integration of machine learning approaches into H&E digital pathology workflows has emerged as a promising strategy to improve TLS detection. HookNet-TLS or other deep learning-based models enable automated identification and quantification of TLS, stratification according to maturation status, and evaluation of their prognostic significance (**Figure 2**) (30, 31).

IHC provides a more reliable method for the identification of TLS compared to H&E. CD20/CD23 double staining coupled with CD23 single staining has been tested on heterogeneous solid tumor samples (**Figure 2**) (29). The selected markers help in the identification procedure, since mTLS always comprise B cells and FDCs. However, conventional IHC is inherently limited by the number of markers that can be assessed simultaneously, and the co-detection of multiple antigens expressed by the same cell type or within the same subcellular compartment may be compromised by steric hindrance, which can impede effective binding of primary antibodies.

Fluorescence-based imaging approaches can help increase the number of TLS-specific features. TLS-specific antibody panels for melanoma tissues have been developed and can be extended to other tumors (32). A complete TLS identification panel should include antibodies against essential lymphoid components such as T cells (CD3), B lymphocytes (CD20), and DCs (DC-LAMP), as well as non-lymphoid components such as PNAd for HEVs (**Figure 2**). However, caution is advised with PNAd and DC-LAMP staining, as their expression has been described in lymphocyte-rich areas without TLS-like organization (33). Moreover, TLS maturation can be assessed using antibodies against GC maturation markers like CD21 and CD23, alongside B-cell activation markers such as AID and Ki67. Additionally, markers like ILT4, FOXP3, CD4, CD8, Eomes, T-bet, GATA3 and RORγt can be included to describe the level of T cells activation and immune suppression (32).

More complex imaging approaches could also be applied for TLS identification (34). The MILAN cyclic method has been used to stain melanoma TME with 38 different antibodies and to identify GC-like areas with high accuracy (35). Hoch et al. also optimized an imaging mass cytometry (IMC) protocol to study melanoma microenvironment by measuring 12 transcripts (encoding for chemokines) and 51 proteins (36). This work revealed that dysfunctional LAG3^+^ CD8^+^ T cells are major source of secreted CXCL13 in the chronically inflamed TME, leading to B-cell recruitment and follicle-like structures formation (36). Additionally, significant recruitment of naïve TCF1^+^ T cells and memory CD45RO^+^ T cells in pre-existent B-cell follicles has been observed, with these cells compartmentalizing to form mTLS (12, 36, 37). IMC offers a strong method for TLS identification, with chemokine staining providing complementary insights into TLS maturity (**Figure 2**). Distinguishing TLS by their maturation status is essential to detect meaningful differences in survival analyses between TLS-positive and TLS-negative groups (37).

Other groups have adopted imaging-independent, molecular biology-based strategies to identify TLS and stratify patients. A TLS-related gene expression risk model, based on the expression levels of CCL8, CXCL13, and IRF4, has been developed and validated in a melanoma cohort (38). This model effectively stratifies patients independently of conventional clinicopathological parameters. Patients classified as low risk typically exhibit a higher density of TLS within their TME. This approach exemplifies the increasing relevance of contemporary TLS-identification methods in the clinical and molecular characterization of melanoma.

Liu et al. employed slide-T-cell receptor (TCR) sequencing to investigate the functional state of T cells within TLS in melanoma metastases (39). Their analysis, based on the sequencing of whole transcriptome and TCRs within intact tissues, revealed that T cells residing in TLS exhibited lower clonal expansion compared to those infiltrating the tumor core. Spatial immune profiling further showed that CD4^+^ T cells were the predominant subset within TLS, whereas exhausted CD8^+^ T cells were progressively enriched closer to the melanoma mass. These findings highlight distinct immune landscapes between TLS and intratumoral regions, suggesting that the antitumor response mediated by TLS may rely more heavily on CD4^+^ T-cell activity than on CD8^+^ T-cell function (39).

Overall, the available tools for TLS identification differ markedly in their readiness for clinical use (**Table 1**). AI-based analysis of H&E slides (HookNet-TLS and, more recently, the YOLOv8 classifier (40)) is closest to translation, reaching >90% accuracy across cancer types with existing pathology infrastructure, although it has not been validated in melanoma. Moreover, cross-institutional standardization and regulatory approval are still missing. IHC, another accessible technique, is limited by its narrow marker panel and the lack of standardized quantification algorithms. On the other side, IMC provides high single-cell multiplexing but remains restricted to specialized centers by cost and low throughput. While imaging-independent methods are under development, their application causes loss of the spatial localization of TLS within the tumor. Similarly, slide-TCR sequencing requires further evaluation and remains cost-prohibitive. Therefore, at the moment, AI-assisted H&E analysis represents the most realistic near-term route to clinical TLS assessment in melanoma. From a workflow perspective, AI-based TLS detection tools operate on whole-slide images compatible with digital pathology scanners already integrated into clinical pathology workflows, requiring no additional hardware infrastructure. Their clinical translation, however, remains contingent on regulatory clearance as Software as a Medical Device (SaMD), with both FDA and EU *In Vitro* Diagnostic Regulation (IVDR) frameworks mandating prospective clinical validation and post-market surveillance, milestones that no TLS-specific classifier has yet achieved.

**Table 1:** TLS identification technologies.

Technique	TLS biomarkers	TLS identification	Estimated cost	Advantages	Limitations	Clinical application	Ref
Hematoxylin-Eosin (H&E) staining	None (morphology-based) • Lymphocyte nuclear morphology • Cellular density • Nuclear volume vs. adjacent tissue	Manual morphological evaluation of lymphoid aggregates; necrotic/ulcerated areas excluded; AI-assisted (Deep Learning): • HookNet-TLS and other DL models enable automated TLS detection and quantification • Maturation stratification	Very low	Most cost-effective; Rapid turnaround; Broad clinical availability; Operator-independent; Scalable to large cohorts	Lower specificity than image-based methods; operator-dependent	yes	(30, 31, 40)
Immunohistochemistry (IHC)	CD20 (B cells) CD23 (FDCs)	CD20/CD23 double staining + CD23 single staining, validated on solid tumor samples	Low	More reliable than H&E; Routinely available; formalin-fixed paraffin embedded (FFPE) compatible	Limited number of simultaneous markers; Steric hindrance can impair co-detection	yes	(29)
Multiplex immunofluorescence (mIF)	CD3, CD20, DC-LAMP, PNAd CD21, CD23, AID, Ki67	Simultaneous spatial mapping of T/B-cell zones, HEVs and mature DCs; Maturation and T-cell activation/suppression profiling; MILAN (38-antibody panel) identifies GC-like areas	Moderate–High	High multiplexing capacity; Combines spatial and phenotypic data; Full maturation profiling	Complex protocol optimization; PNAd/DC-LAMP also detected outside TLS-like areas	yes/no	(32–35)
Imaging Mass Cytometry (IMC)	CD20, CD4, CXCL13, CD8, PD-1, TCF7	Single-cell spatial profiling of the tumor microenvironment; Chemokine staining informs TLS maturity	High	Highest simultaneous marker capacity; Single-cell spatial resolution; Combined protein/RNA detection	Technically demanding and expensive; Low throughput; Specialized centers only	no	(36)
Gene expression signature (GES)	CCL8, CXCL13, IRF4 (TLS-related risk model)	Gene expression-based risk model stratifies patients independently of clinicopathological parameters; low-risk patients show higher intratumoral TLS density	Low–Moderate	Imaging-independent; Scalable to large cohorts; FFPE-compatible; Validated prognostic value in melanoma	No direct spatial/morphological confirmation of TLS; Indirect identification only	yes	(12, 38)

## TLS in primary melanomas

TLS have been identified in primary cutaneous melanomas, particularly within the superficial spreading and nodular subtypes, where their presence positively correlates with increased Breslow thickness and they are more frequent in primary melanomas of the trunk, head, and neck compared to those arising on the limbs (37).

Phenotypic tissue studies have shown that primary melanomas harbor TLS with an early immature phenotype, lacking BCL6^+^-GC. iTLS in primary tumors are predominantly located intratumorally, typically within 1 mm of the invasive margin (33). However, no statistically significant correlation was found between TLS density, size, or location in primary tumors and major clinical parameters (33), indicating that mere spatial and quantitative TLS features in primary melanomas alone are insufficient to predict clinical outcomes.

A more recent study, conducted on a larger cohort of samples, has shown that lymphoid aggregates formation in primary melanoma lesions is positively associated with tumor-free regional lymph nodes (41). Interestingly, deeper histological evaluation, based on immunofluorescence detection of CD8, CD20, CD21, and PNAd, confirmed that most of the identified lymphoid aggregates are indeed iTLS (41).

In primary desmoplastic melanoma (DM), a rare melanoma subtype characterized by dense fibrous stroma, TLS are less frequent and tend to be smaller in diameter compared to those in non-desmoplastic melanoma (NDM). DM-TLS are typically located intratumorally, while NDM-TLS are more often found in peritumoral regions (42). Despite their reduced size and number, DM-TLS exhibit a higher density of CD8^+^ T cells, CD20^+^ B cells, and proliferating lymphocytes (Ki67^+^) than NDM-TLS (43), suggesting a more mature immunological state. Interestingly, the proliferation rate of lymphocytes within TLS positively correlates with that of lymphocytes within the tumor core in DM. This correlation is absent in NDM, possibly reflecting differences in TLS maturity between these two histological subtypes (43). These observations suggest that TLS maturity and the extent of lymphocyte proliferation may hold greater prognostic value than TLS number or size alone.

Digital spatial profiling proteomic studies on primary DM revealed enrichment of CD20^+^ B cells within lymphoid aggregates compared to adjacent tissue, supporting the presence of TLS (44). The same analysis uncovered substantial expression of the immune checkpoint molecules CTLA4 and LAG3, both within and surrounding TLS-like aggregates. Notably, the expression of these immunosuppressive markers was higher in the broader TME than within the aggregates themselves, suggesting that TLS-like structures may maintain a relatively more immunologically active state compared to the surrounding tumor milieu (44).

In primary acral melanoma (AM), intratumoral iTLS are more frequently observed in earlier-stage disease (stage I–II) than in stage III tumors and correlate with improved OS (45). Beyond their prognostic association, intratumoral TLS were linked to a distinct TME composition characterized by increased CD8^+^ T-cell infiltration. On the other side, peritumoral TLS correlated with higher CD68^+^ macrophage infiltration, suggesting that TLS localization may reflect fundamentally different immune contextures within the same tumor. Transcriptomic profiling further demonstrated that TLS maturity is independently associated with both overall and disease-free survival (45).

The observed variation in TLS prevalence across melanoma subtypes likely reflects two interacting determinants: tumor immunogenicity (neoantigen burden and mutation type) and stromal architecture. DM exhibits a higher density of TLS than NDM. This enrichment can be partly attributed to DM elevated TMB and consequent neoantigen load, which generate stronger local immune stimulation and promote TLS neogenesis (46, 47). Concurrently, the dense, fibrotic stroma typical of DM may concentrate infiltrating immune cells and provide the collagenous and stromal scaffold necessary for TLS formation (48). Indeed, CCL19^+^ stromal cells in the TME have been implied in TLS formation in CRC, an event that could occur also in melanoma (48).

Conventional cutaneous melanomas show intermediate TLS frequencies, consistent with a moderate TMB and a stromal environment that is variably permissive to immune cell recruitment and organization (47). The low TMB of AM, in contrast, could produce a reduced neoantigen repertoire and a comparatively sparse tumor immune infiltrate insufficient to sustain TLS formation (49).

The TMB–TLS relationship in melanoma is further supported by a TLS-related prognostic model in which the low-risk group showed higher TMB and correspondingly higher OS (38).

Overall, while these mechanistic hypotheses are concordant with current data, larger systematic and spatially resolved studies are needed to quantify subtype differences and to dissect how neoantigen quality, stromal composition, and local cytokine/chemokine milieus cooperate to drive TLS biogenesis in melanoma.

In conclusion, the current findings on primary melanoma samples indicate that the prognostic weight of TLS is not carried by their presence alone, but by the quality of the immune response they sustain, shaped by their intratumoral localization, structural maturity, and the immune contexture they engender. This has direct implications for how TLS should be assessed in future studies, shifting the analytical focus from enumeration toward functional and spatial characterization.

## TLS in melanoma metastasis

TLS are more frequent in melanoma metastasis than in primary tumors, where they show higher maturity and have a preferential peritumoral localization (33, 41). As melanoma progresses from locoregional to distant, visceral metastasis, there is an increased frequency of mTLS, particularly those containing BCL6^+^ GC (33). These spatial variations in TLS distribution and maturation may contribute to heterogeneous antitumor immune responses and could reflect different clinical responses to immunotherapy. In metastatic lesions, mTLS are reported more frequently in the adipose tissue surrounding the tumor nest than within the tumor parenchyma itself (41). This preferential peritumoral localization raises the possibility that adipokines contribute to TLS maturation and GC formation. This hypothesis is supported by the evidence that adiponectin-stimulated B cells upregulate genes involved in immunoglobulin synthesis, including *Ighg2b*, *Ighg2c*, *Jchain*, and *Iglc1* (41). Whether adiponectin signaling within iTLS can actively drive their maturation into fully functional GC-bearing structures remains an unresolved question.

Despite the growing body of evidence on TLS in melanoma metastases, no site-specific predominance has been established, with TLS frequency appearing comparable across metastatic locations in most cohorts (12, 33, 41). However, this apparent uniformity may reflect the limitations of available studies rather than true biological equivalence. *Werner and colleagues* compared TLS presence and maturation between visceral and brain metastases. They found a complete absence of TLS in the latter, albeit in a very small brain metastasis cohort (33). This observation raises the possibility that organ-specific metastatic niches differ in their capacity to support TLS formation, localization, and maturation, yet systematic site-stratified analyses in melanoma remain lacking. Indirect support for this hypothesis comes from a study comprehensively profiling the immune microenvironment across distinct anatomical sites, demonstrating that melanoma metastatic location significantly shapes immune infiltration and cellular composition (50), prerequisites for TLS neogenesis. A recent pan-cancer study demonstrated that TLS are neither spatially uniform nor immunologically equivalent across cancer types and anatomical locations (40), underscoring the need for systematic, site-resolved TLS characterization in melanoma metastases.

TLS are not merely bystander lymphoid aggregates; rather, they represent spatially organized hubs of ongoing adaptive immune activity. In melanoma metastasis, intratumoral TLS positively correlate with CD8^+^ T cell infiltration beyond the TLS boundaries and are associated with superior OS compared to peritumoral TLS, highlighting the prognostic importance of TLS localization (12). Within or adjacent to TLS, particularly those harboring highly proliferative CD20^+^ B cells, T cell populations are skewed toward CD4^+^ T helper cells with high BCL2 expression and relatively poor CD8^+^ T cell representation (12). These results suggest that TLS may sustain local T cell survival while the cytotoxic response is predominantly executed in the surrounding parenchyma.

The functional state of B cells within TLS could influence the entire TME. TLS enriched in CD20^+^ AID^+^ B cells, a phenotype indicative of active antibody affinity maturation, are associated with favorable clinical outcomes in metastatic melanoma, whereas TLS composed of CD20^+^ CD21^+^ B cells correlate with poorer prognosis (51). This dichotomy suggests that the capacity of intratumoral B cells to generate high-affinity antitumor antibodies may be a critical determinant of effective immune-mediated tumor control (52).

The isotypic identity of TLS-derived antibodies adds a further layer of complexity. While IgG is the predominant antibody class produced by B cells within TLS, IgA has also been identified as a relevant secreted isotype in the melanoma TME (53). Unlike IgG, IgA does not engage complement-dependent cytotoxicity or antibody-dependent cellular cytotoxicity, raising questions about its functional contribution to antitumor immunity. IgA class switching within TLS may instead reflect a state of peripheral immune tolerance, analogous to mechanisms observed in secondary lymphoid organs (10), a hypothesis that needs further investigation.

Finally, while TLS-positive melanoma metastases exhibit increased intratumoral lymphocytic infiltration (51), it remains unresolved whether lymphocyte proliferation within TLS is functionally coupled to that observed in the broader intratumoral compartment. Clarifying this relationship will be essential to fully establish TLS as a reliable surrogate of hot TME status and to determine whether TLS-derived immune activity directly drives, or merely reflects, the antitumor response (**Table 2**).

**Table 2:** TL S identification in melanoma and clinical significance.

Primary/metastatic	Stage	Melanoma histotype	TLS localization	TLS identification system	Maturation status	B cell subsets	TLS-related parameter	Clinical value	Patient cohort and statistics	Description	Ref
PRIMARY MELANOMA	I - II	SSM, nodular	Not described	Histology/IHC: CD20+ B-cell follicles ± CD21+ FDC network, CD45RO+ T cells, MECA-79+ HEV	Not defined	Morphological follicular B cells with partial AID expression	TLS abundance	Prognostic	147 patients; p = Not defined	TLS presence and Breslow thickness improve prognostic stratification	(37)
	I - II	Not defined	Mostly peritumoral	H&E + multiplex immunofluorescence + transcriptomics	iTLS	Not described	Immature TLS presence	Prognostic	195 patients; p = 0.002	Immature TLS presence correlates with tumor-negative regional lymph node	(41)
	I-II	SSM, nodular	Within 1mm from intratumoral compartment	7-color mIHC (CD20, CD4, CD21, CD23, CXCL13, BCL6, DAPI); eTLS/pTLS/sTLS staging by FDC network (CD21/CD23) and BCL6 GC	iTLS	BCL6 negative	none	none	103 patients; p = Not defined	TLS mostly intratumorally, no clinical correlation described	(33)
	II	Desmoplastic	intratumoral	GeoMx digital spatial proteomic (DSP) profiling of histologically defined LA	Not defined	Not defined	LAG-3, CD8, CD8, PTEN, OX40L, SMA	Prognostic	45 patients; p(LAG-3) = 0.035, p(CD8) = 0.046, p(OX40L) = 0.044, p(PTEN) = 0.032	Higher LAG-3 expression associated with worse overall survival. Higher expressions of CD8, PTEN, OX40L, and SMA associated with worse recurrence-free survival	(44)
	I, II, IIl	Acral	Stage I-II more intra-TLSs	H&E+mIF (CD20, CD21, CD23) for eTLS/pTLS/sTLS	Stages I-II more iTLS	Not defined	TLS presence	Prognostic	46 patients; p = 0.032	TLS presence and their intratumoral localization positively correlate with OS	(45)
METASTATIC MELANOMA	III	Not defined	Not defined	Bioinformatic TLS-related gene signature (LASSO/Cox risk model); no histological assessment	Not defined	Not defined	TLS-related gene risk model	Prognostic	454 patients; p = 7.865e-06	Low risk patients significantly correlate with improved OS	(38)
	III	Not defined	Not defined	Bioinformatic TLS-related gene signature (LASSO/Cox risk model); no histological assessment	Not defined	Not defined	TLS-related gene risk model	Predictive	454 patients; p < 2.22e-16 (CTLA4+PD-L1+) p = 9e-12 (CTLA4- PD-L1+)	Improved ICI responses for low risk patients	(38)
	III- IV	Not defined	Multiple locations, mostly peritumoral	H&E+multiplex immunofluorescence+ transcriptomics	iTLS and mTLS	Not defined	TLS presence	Prognostic	83 patients; p = Not defined	TLS^+^ patients are characterized by improved RFS and OS after metastasectomy	(41)
	III- IV	SSM, nodular	Multiple locations	H&E identification+IHC+GeoMx spatial proteomics+immune gene signature	iTLS and mTLS	Ki67+ B cells in mTLS	TLS localization	Prognostic	33 patients; p = 0.1 (trend)	Intratumoral TLS positively correlate with longer OS compared to peritumoral TLS	(12)
	III- IV	SSM, nodular	Multiple locations	H&E identification+IHC+GeoMx spatial proteomics+immune gene signature	iTLS and mTLS	Ki67+ B cells in mTLS	TLS density	Predictive	186 patients; p < 0.05	Higher number of TLS gene signature correlates with improved response to ICI	(12)
	III- IV	SSM, nodular	Not defined	Multi-omic: RNA-seq deconvolution (MCP-counter), CyTOF B-cell phenotyping, BCR sequencing	Not defined	naive, memory, plasmacells (whole tumor)	TLS to tumor area ratio	Predictive	19 patients; p = 0.037	TLS wider than tumor correlate with improved response to neoadjuvant ICI	(61)
	III- IV	SSM, nodular	Not defined	Multi-omic: RNA-seq deconvolution (MCP-counter), CyTOF B-cell phenotyping, BCR sequencing	Not defined	naive, memory, plasmacells (whole tumor)	B cell receptor diversity	Predictive	21 patients; p < 0.001	B cell receptor diversity high in ICI responders	(61)
	III- IV	Not defined	Not defined	Multiplex IHC; eTLS/pTLS/sTLS maturation staging with quantification of CD20+AID+ and CD20+CD21+ B-cell subsets, CD8+EOMES+ T cells	iTLS and mTLS	CD20+ AID+ B cells	B cell somatic hypermutation	Prognostic	64 patients; p = 0.03	The proportion of intra-TLS B cells performing somatic hypermutation positively correlates with improved OS	(51)
	III- IV	Not defined	Not defined	Multiplex IHC; eTLS/pTLS/sTLS maturation staging with quantification of CD20+AID+ and CD20+CD21+ B-cell subsets, CD8+EOMES+ T cells	iTLS and mTLS	CD20+ AID+ B cells	CD20^+^ CD21^+^ B cells	Prognostic	64 patents; p = 0.02	Numerosity of this B cell subset within TLS negatively correlates with OS	(51)
	III- IV	Not defined	Not defined	Multiplex IHC; eTLS/pTLS/sTLS maturation staging with quantification of CD20+AID+ and CD20+CD21+ B-cell subsets, CD8+EOMES+ T cells	iTLS and mTLS	CD20+ AID+ B cells	CD20^+^ AID^+^ B cells	Prognostic	64 patients; p = 0.03	Numerosity of this B cell subset within TLS positively correlates with OS	(51)
	III- IV	SSM, nodular, acral	Not defined	Meta-analysis pooling heterogeneous H&E/IHC criteria from included studies	Not defined	Not defined	Presence or higher TLS density	Prognostic	6 studies; p < 0.001	Positively correlates with OS and PFS of ICI treated patients	(58)
	IV	Not defined	Not defined	12-chemokine gene expression signature	Not defined	Not defined	12 chemokine gene signature	Prognostic	120 patients; p = 0.008	Gene signature positively correlates both with TLS presence and improved OS	(60)

As described above, distinct tumor genetic contexts could influence TLS formation and maturation (40, 54). In melanoma, driver mutations such as BRAF and NRAS may directly modulate TLS neogenesis, although direct experimental evidence is lacking. Immune profiling studies showed that treatment-naïve BRAF-mutant melanoma harbors a distinct TME compared to BRAF wild-type, with increased B cell and CD4^+^ T cell infiltration alongside reduced CD8^+^ T cells, consistent with TLS-positive tumors (55). Elevated B cells in BRAF-mutant metastatic melanoma are associated with improved survival (55). In a separate study, gene expression profiles revealed that a high T-cell/low B-cell signature associates with favorable outcomes in patients undergoing dabrafenib plus trametinib treatment, whereas a high T cell/high B-cell signature correlates with poorer survival (56). In addition, preclinical studies have shown that targeted therapies can directly impair immune cell function, potentially affecting TLS. In a DC–T-cell co-culture system, combined vemurafenib and cobimetinib treatment reduced the expression of T-cell activation markers (CD25 and CD69), suppressed spontaneous and antigen-specific T-cell proliferation, decreased IL-2, IFN-γ, and TNF-α production. This treatment also impaired DC maturation, limiting CD80, CD86, and CCR7 expression (57).

Collectively, these findings suggest that BRAF mutational status shapes immune cell recruitment and organization within the TME, and that targeted therapies could alter the functionality of TME immune architecture, including TLS. This may have direct implications for TLS biology and for the rational sequencing of targeted and immunotherapeutic strategies. However, these observations remain preliminary, and dedicated studies systematically interrogating the relationship between melanoma driver mutations, TLS formation and composition, and the immunomodulatory effects of targeted therapy on TLS biology, are needed.

Most of the literature has instead focused on TLS in metastatic melanoma and ICI responses (**Table 2**). Intratumoral TLS formation and density are positively correlated with both overall and progression-free survival in ICI-treated patients (58, 59). These findings emphasize the need for a standardized TLS maturity staging system and suggest that disease-specific TLS classification criteria may be essential, given the diverse features of TLS across different tissues and tumor types.

Initial studies in stage IV melanomas, showed that the presence of lymph node-like structures (presumably TLS) in metastatic lesions correlated to the expression of a 12-chemokine gene expression signature (GES), encompassing CCL2, CCL3, CCL4, CCL5, CCL8, CCL18, CCL19, CCL21, CXCL9, CXCL10, CXCL11, and CXCL13 (60). In the same study, TLS were enriched in B cells and contained few CD4^+^ FOXP3^+^ regulatory T (Treg) cells, suggesting predominantly an antitumor function. Notably, higher TLS-GES score positively associates with improved OS, highlighting its potential utility in refining prognostic stratification for melanoma patients (60).

Bulk RNA-seq analyses have revealed that several immune-related signaling pathways are enriched in melanoma patients who respond to ICI therapy compared to non-responders. These include TCR signaling, antigen processing and MHC-mediated presentation, Th1/Th2 cell differentiation, and co-stimulatory signaling involved in T-cell activation (61). Such transcriptional signatures suggest active B–T cell crosstalk and highlight the potential role of mTLS as key sites for productive lymphocyte interactions that enhance antitumor adaptive immunity. Indeed, a B cell-related transcriptional signature was enriched in patients responding to neoadjuvant ICI therapy, both at baseline and during early treatment. This signature includes genes involved in plasma cell differentiation and immunoglobulin synthesis (MZB1, JCHAIN, IGLL5), immune regulation (BTLA), inflammation and antigen presentation (IRF1, IFNG), and memory B-cell activation (FCRL5) (61). Notably, this signature can be detected as enriched independently of metastasis location, whether subcutaneous or in draining lymph nodes. ICI responders consistently exhibit higher CD20^+^ B-cell density, increased TLS abundance, and a greater TLS-to-tumor area ratio, particularly in early on-treatment biopsy specimens (61). Moreover, ICI responders display significantly higher B-cell receptor diversity compared to non-responders, suggesting that TLS may contribute to an active and diversified humoral antitumor response (61).

TLS-related gene risk models (computational tools, that stratify patients according to the expression profiles of TLS-associated genes) have demonstrated potential predictive value in melanoma (12). Patients classified as low risk by these models exhibit higher response rates to ICI therapy compared to those in the high-risk group (12, 38). These findings suggest that pre-existing TLS may enhance ICI efficacy, while immunotherapy itself could further promote TLS development and function, indicating a potentially bidirectional relationship that warrants further investigation.

Although metastatic melanomas generally display lower densities of HEVs compared to primary tumors, these specialized vessels remain detectable and functionally relevant (62).

In stage III and IV melanoma, increased density of HEVs positively correlates with clinical responsiveness to combined ICI therapy (anti-PD-1 plus anti-CTLA4), as well as with improved overall and progression-free survival (63). HEVs are essential for facilitating lymphocyte recruitment into both secondary lymphoid organs and TLS, thereby supporting the progressive maturation of local immune structures and the establishment of long-term antitumor immunity. These observations highlight the importance of assessing TLS formation during and after ICI treatment in prospective studies. Supporting this notion, high pre-treatment expression of complement factor C2 in the TME has been associated with increased TLS formation and prolonged OS in metastatic melanoma patients. Moreover, C2 expression increases following anti-PD-1 (nivolumab) therapy, suggesting that ICIs may actively remodel the TME to favor TLS neogenesis (64).

Taken together, existing studies in metastatic melanoma have established a clear predictive and prognostic role for TLS; however, significant gaps remain. These include the absence of a consensus framework for TLS identification and maturation staging, and a limited understanding of why a subset of TLS-positive patients fail to respond to targeted therapy or ICI.

## TLS as immunosuppressive niches

Distinct T- and B-cell states coexist within TLS, suggesting that not all TLS support effector programs. Indeed, some TLS can instead favor immunoregulatory functions.

In NSCLC, TLS-infiltrating Tregs suppress the proliferation and cytokine secretion of CD4^+^ conventional T cells, limiting TLS antitumor activity (65). In ovarian cancer, mature TLS harbor not only CD8^+^ effector T cells but also a TIM3^+^ PD1^+^ exhausted subset associated with poor response to ICI (66). Similarly, in melanoma metastases, elevated densities of EOMES^+^ exhausted T cells correlate with worse OS (51).

A parallel pattern of immunosuppressive heterogeneity exists within the B-cell compartment. Increased densities of CD20^+^ CD21^+^ B cells (described as anergic or exhausted B cells (67)) predict poor OS in melanoma metastases (51). In bladder cancer, IGLL5^+^ B cells bind HEVs and drive TLS disassembly, thereby impairing responses to immunotherapy (68). Regulatory B cells (Bregs), functionally defined by IL-10 secretion (69), could represent a further layer of TLS heterogeneity, however they have been understudied (70).

These findings support a model in which TLS can be structurally intact yet functionally tolerogenic niches shaped by Breg, Treg, and exhausted T-cell states. Consistent with this, TLS maturity alone does not guarantee the presence of effective antitumor immune responses and ICI-responsive phenotypes.

## Modulating TLS formation: novel immunotherapeutic avenue

The evaluation of TLS is influenced by both inter- and intra-patient microenvironment variability; however, elucidating the mechanisms that regulate (and potentially allow therapeutic manipulation of) TLS formation and function could provide valuable strategies to enhance immunotherapy efficacy.

### Preclinical studies

TLS induction has been performed by intratumoral administration of low-dose ADU-S100, a STING pathway agonist (71). In subcutaneous B16-F10 melanomas, ADU-S100 injection induced permeable HEVs neogenesis and enhanced tumor infiltration of CD54^high^ CD86^high^ CCR7^high^ PD-L1^+^ CD11c^+^ mature DCs and CD3^+^ T cells, most of which are CD8^+71^. ADU-S100 also enhances TLS neogenesis-related factors, such as LTα_3_, TNF-α, and IL-36β, from mature DCs. Moreover, it upregulates CCL19, CCL21, LTα-β, and LIGHT expression in the TME, further promoting tumor-associated TLS formation. However, ADU-S100 administration did not induce CXCL13 expression in the TME, leading to a lack of evident B-cell zones in the newly formed TLS. Despite their immature phenotype, these TLS can recruit peripheral effector lymphocytes (71), suggesting that STING agonists alone are insufficient to induce fully functional TLS. Moreover, ADU-S100 faces significant challenges due to its poor bioavailability upon intratumoral or systemic injection and dose-dependent toxicities, necessitating low dose administration.

Cheng and colleagues sought to overcome these limitations by constructing M1 macrophage-derived extracellular vesicles loaded with ADU-S100, creating the ADU-S@M1 (72). Notably, in the B16-F10 melanoma model, these vesicles promoted the polarization of tumor-associated macrophages toward a M1 phenotype and the maturation of DCs.

ADU-S@M1 treatment also remodels the stromal compartment, and fibroblasts in the TME acquire LTo-like functions. This is accompanied by sustained overexpression of key chemokines involved in TLS neogenesis, ultimately leading to TLS formation and promoting *in situ* antitumor immune responses. Mice treated with ADU-S@M1 exhibited enhanced suppression of B16-F10 tumor growth and survival and responded more effectively to anti-PD-1 therapy. These findings are consistent with evidence supporting improved ICI efficacy in TLS-positive patient subgroups (72).

Additionally, recombinant interleukins administration may promote lymphangiogenesis and TLS formation, although this strategy is mainly impeded by short half-life of administered interleukins, and dose-dependent toxicity. In melanoma preclinical model, IL15-expressing oncolytic adenovirus (IL15-Ad) triggered both lymphatic vessel and TLS formation (73). IL15-Ad induced blood and lymphatic vessels maturation, increasing lymphocyte recirculation between the TME and lymphoid organs. IL15-Ad also stimulated the production of LTβR agonists, CCL19, CCL21 and CXCL13 in the TME, thereby promoting TLS formation. However, these induced TLS appeared to be immature and not completely functional (73). In this context, mature DCs foster lymphatic vessel maturation and TLS formation. Indeed, IL15-Ad stimulates the STING pathway in these mature DCs, increasing the expression of LTα_3_, CXCL10, IL-6 and IFN-β. As a matter of fact, experimental inhibition of the STING-TBK1 complex resulted in reduced DCs-T cells crosstalk and diminished TLS formation (73).

Several other strategies have successfully promoted TLS neogenesis in preclinical models beyond melanoma setting.

Engineered exosomes co-delivering phosphorylated STING, an anti-CD47 nanobody, and the autophagy inhibitor BafA1 remodeled the glioma microenvironment by inducing CCL19, CXCL9, and CXCL13 expression, suppressing TGF-β, and promoting DC activation and M1 macrophage polarization (74). These changes supported sustained antigen presentation and chemokine gradients, both critical for TLS development. Continuous cytokine signaling also proved essential, as LIGHT-expressing *Salmonella typhimurium* induced both TLS formation and maturation in CRC through TNF receptor signaling (75). Similarly, slow-releasing polymer systems delivering CCL19, CCL21, CXCL12, CXCL13, LT-α_1_β_2_, and soluble RANKL substituted for LTo function, facilitating TLS formation (76). When embedded in polyethylene glycol/collagen macroporous scaffolds and implanted in tumor-free models, these systems supported plasma cell differentiation and antigen-specific IgG production, suggesting functional B-cell zone development (76, 77). Moreover, biomaterial-based approaches can be exploited to promote lymphocyte spatial organization. While exerting antitumor effects via photothermal activity and reactive oxygen species generation, photoresponsive covalent organic frameworks (TPDA-ViBT-COFs) induced TLS formation in CRC when combined with laser photoactivation (78).

Adipose tissue-derived mesenchymal stromal cells conditioned with TNF-α and incorporated into 3D lymphocyte co-cultures generated organoid systems with spatial immune compartmentalization resembling iTLS (79). These organoids were also encapsulated in autologous fibrin glue and implanted into murine fat depots, becoming vascularized, infiltrated by host immune cells, and remodeled, though full TLS maturation was not achieved (79). Human induced pluripotent stem cell-derived HEV organoids — exhibiting high expression of LTi markers including LTβR, IL-7, CCL19, and CCL21 — have demonstrated antigen presentation capacity, lymphocyte recruitment, and adaptive immune priming in NOD/SCID models (80, 81). HEV organoids progressively acquired structural and functional resemblance to TLS, inhibiting progression of engrafted tumors (81).

### Clinical studies

Immunomodulating combinatorial strategies may represent a powerful tool for TLS induction. A phase Ib clinical trial in patients with resectable advanced AM (stage III and IV) demonstrated that neoadjuvant therapy combining the oncolytic virus OrienX010 with the anti-PD-1 antibody toripalimab effectively promoted tumor regression over a 24-month follow-up period (82). Notably, patients responding to the treatment exhibited a higher density of TLS and greater lymphocytes infiltration in tumor beds compared to non-responders (82). Liquid biopsy and inflammatory protein profiling revealed that all treated individuals had significantly elevated circulating levels of IFN-γ, granzyme-A, IL-12, and CXCL10 (82). These findings are consistent with additional reports describing herpes simplex virus type 1 (HSV-1)-derived oncolytic viruses that increase the number of TCF1^+^ CD8^+^ tumor infiltrating lymphocytes through cytokine and chemokine upregulation. In particular, the CXCL10 - CXCR3 signaling axis appears to play a critical role in remodeling the TME and facilitating TLS neogenesis (83). Combination therapy with HSV-1-based oncolytic viruses and anti-PD-1 antibodies has been correlated with improved preclinical and clinical outcomes (83, 84).

Peptide vaccines have demonstrated the capacity to induce TLS formation in humans (85). The time gap between vaccine administrations and the number of vaccinations on the same site are essential parameters for effective lymphoid aggregates and TLS formation. The formulation composed of 12 different melanoma antigens, poly-ICLC (TLR agonist) and incomplete Freund’s adjuvant seems to be one of the best combinations for increasing the 12-chemokine gene signature referred to TLS neogenesis (60, 86).

Radiation therapy is increasingly recognized as a powerful modulator of the solid TME, with the capacity to promote both the formation and maturation of TLS (87–89).

TLS induction can be modulated by optimizing both radiation dose and irradiation scheduling. High-dose radiotherapy is typically associated with general immunosuppression, which includes impaired T-cell infiltration and proliferation, reduced cytokine production, and diminished natural killer cells and T lymphocytes effector functions, alongside increased recruitment of immunosuppressive myeloid-derived suppressor cells (88, 90). In contrast, low-dose fractionated radiotherapy combined with PD-1 blockade has been shown to enhance T-cell infiltration and activation within the TME, resulting in improved local and systemic tumor control. This combinatorial approach promotes the priming of endogenous antigen-specific T and B cells, strengthening both cellular and humoral antitumor immunity, particularly in melanoma models (91). However clinical data in melanoma patients are currently unavailable.

Several ongoing clinical trials are evaluating novel therapeutic targets to promote TLS induction across a variety of solid tumors (92). Among these, CD40 and semaphorin 4D have emerged as particularly promising candidates for melanoma immunotherapy (92, 93). Nevertheless, further studies are needed to determine whether CD40 agonists primarily enhance the maturation of pre-existing TLS or are capable of inducing TLS neogenesis in TLS-negative lesions. Likewise, the combination of ICIs and semaphorin 4D inhibitor remains to be optimized to effectively convert TLS negative TME into mTLS-rich TME, thereby improving responses in patients who are refractory to current immunotherapies.

Therapeutic strategies that induce TLS formation, including STING agonists, oncolytic viruses, cytokine modulation, and optimized radiotherapy, represent a convergent approach to reshaping the immunosuppressive TME. The synergistic potential of combining these modalities with ICIs offers substantial promise for improving clinical outcomes in melanoma. Further research will be essential to translate these preclinical and early clinical successes into more broadly applicable and effective clinical interventions.

## Conclusions and future directions

TLS have emerged as critical immune niches across several inflammatory diseases. In oncology, TLS are increasingly recognized as dynamic components of the TME, frequently associated with improved clinical outcomes and enhanced responses to ICI. Despite growing enthusiasm, several critical points still need to be addressed before TLS can be reliably leveraged for clinical decision-making across solid tumors.

1. Standardized TLS detection. Robust, standardized methods are needed to detect TLS and understand their role as prognostic biomarkers.
2. Determining whether TLS actively drive antitumor immunity. Different features, including composition, maturation status, and spatial organization, shape TLS heterogeneity, and influence their capacity to support effective immune responses. Identifying biomarkers that reliably distinguish functional, immunostimulatory TLS from dysfunctional or immunosuppressive counterparts remains a pressing need.
3. Identify TLS-associated immunosuppressive mechanisms. While multiple studies point out TLS heterogeneity, systematic research into their immunosuppressive mechanisms remains scarce.
4. Therapeutic exploitation of TLS. If TLS can be induced or reprogrammed to support durable antitumor immunity, they could represent a novel and powerful strategy to complement existing immunotherapies. Achieving this goal will require a deeper mechanistic understanding of TLS initiation, maintenance, and function, as well as careful consideration of context-dependent effects across cancer types and anatomical sites.
